# Coccidioidomycosis in a State Where It Is Not Known To Be Endemic — Missouri, 2004–2013

**Published:** 2015-06-19

**Authors:** George Turabelidze, Ravi K. Aggu-Sher, Ehsan Jahanpour, C. Jon Hinkle

**Affiliations:** 1 Missouri Department of Health and Senior Services

During 1998–2012, coccidioidomycosis cases increased nationally nearly eightfold (1). To describe the epidemiology of coccidioidomycosis in Missouri, a state without endemic coccidioidomycosis, coccidioidomycosis surveillance data during 2004–2013 at the Missouri Department of Health and Senior Services were retrospectively reviewed. The incidence of reported coccidioidomycosis increased from 0.05 per 100,000 population in 2004 to 0.28 per 100,000 in 2013, with cases distributed throughout all regions of Missouri. Persons aged >60 years were most affected. In cases in which patients had disease manifestations, the most common were pneumonia (37%) and influenza-like illness (31%). Nearly half (48%) of patients had traveled to an area where coccidioidomycosis is endemic, whereas approximately one-quarter (26%) of patients did not report such travel. Those with history of travel to endemic areas were significantly more likely to receive a diagnosis by positive culture or polymerase chain reaction (PCR) testing, compared with those without a history of travel to endemic areas, who were more likely to receive a diagnosis by serological tests. Additional studies will be required to ascertain whether truly endemic cases exist in Missouri.

Coccidioidomycosis, or Valley Fever, is a systemic disease caused by the fungus *Coccidioides*, which is endemic to the southwestern United States, Mexico, and Central and South America (2). This fungus normally resides in soil, but airborne spores can cause infection if inhaled. Sixty percent of infections are asymptomatic and do not come to medical attention (3). Manifestations range from influenza-like illness to pneumonia, lung nodules, and disseminated infections. Laboratory tests typically used for coccidioidomycosis diagnosis include complement fixation, immunodiffusion, enzyme immunoassay, culture, histopathology, and PCR. In most patients, infection is mild and resolves without specific antifungal treatment. Azole antifungals are the most commonly used drugs in patients who require treatment (4).

During 1998–2011, among all coccidioidomycosis cases reported to CDC from 28 states and the District of Columbia, 97% were from Arizona and California, 1% from states where coccidioidomycosis is uncommon, and <1% from states where it is not endemic (4). In areas where coccidioidomycosis is endemic (excluding Texas), the age-adjusted incidence of reported cases increased sevenfold from 5.3 cases per 100,000 population in 1998 to 42.6 per 100,000 in 2011 (5). In states where it is not endemic, in 2011, 240 coccidioidomycosis cases were reported, compared with only six in 1998 (1).

Coccidioidomycosis surveillance data during 2004–2013 at the Missouri Department of Health and Senior Services were retrospectively reviewed. Only cases meeting the definition of a confirmed case of coccidioidomycosis as defined by CDC’s National Notifiable Diseases Surveillance System and the Council of State and Territorial Epidemiologists were included (6).

Patients with known travel history were categorized into three groups: 1) those who had not traveled to an area where coccidioidomycosis is known to be endemic, 2) those who had traveled at any time to an area where it is endemic, and 3) those who had recently traveled to an area where the disease is endemic. Recent travel was defined as travel associated with the experience of symptoms either during travel or within 21 days of leaving the endemic area. Poisson regression analysis was used to model the incidence of coccidioidomycosis reported in Missouri.

A total of 93 confirmed coccidioidomycosis cases were reported during the study period (Table). Disease incidence increased from 0.05 per 100,000 population in 2004 to 0.28 per 100,000 in 2013 (p<0.001) (Figure 1). The median age of patients was 58 years (range = 19–94 years). Among 51 (55%) patients with a known symptom onset date, median time to diagnosis was 25 days (range = 3–304). Fungal culture (31%) and complement fixation (30%) were the most common diagnostic tests. Forty-three (46%) patients required hospitalization (five in intensive care). Among 29 patients who received antifungal drugs, 14 were treated as outpatients and 15 were inpatients. Fluconazole was the most used antifungal drug (20% of patients). Eight (8.6%) of the 93 patients died: three deaths were attributed to coccidioidomycosis, three to other illnesses, and the cause of death in the remaining two patients was not reported.

Mapping of cases by residence at the time of diagnosis revealed that patients, with or without travel to an area where coccidioidomycosis is endemic, were distributed throughout all regions of the state (Figure 2). Forty-five patients (48%) traveled to an area where the disease is endemic, 24 (26%) did not, and the travel history for the remaining 24 (26%) was unknown. Among the 45 patients with travel to an area with endemic disease, 19 had recent travel, 20 had travel that was not recent or had a history of residence in an area with the disease, and six had travel timelines that could not be exactly established. The proportion of patients receiving a diagnosis by positive coccidioidomycosis culture or PCR was significantly higher in patients who had traveled (21 of 45) compared with those who had not (4 of 24) (p = 0.018). Overall, among 24 patients without a history of travel, 11 received a diagnosis only on the basis of positive immunoglobulin M or qualitative enzyme immunoassay or immunodiffusion tests, and four received a diagnosis on the basis of positive coccidioidomycosis cultures (all of the latter were immunocompetent). Seventeen (18%) patients were immunocompromised, eight of whom had a history of travel to an area where coccidioidomycosis is endemic.

## Discussion

The increase in the incidence of reported coccidioidomycosis in Missouri from 2004 through 2013 was statistically significant and substantial. The increase is consistent with the national trend of increasing incidence of coccidioidomycosis that includes states with and without endemic disease (1). Mapping of cases with and without a history of travel to areas with endemic disease revealed that cases were occurring in all regions of Missouri.

One explanation for the increase in reported cases could be that coccidioidomycosis became a reportable condition in Missouri in 2003. In comparison, after coccidioidomycosis became reportable in Arizona in 1997, the reported incidence increased from 21 per 100,000 in 1997 to 91 per 100,000 population in 2006 (7). An additional contributing factor could be an increased awareness among health care providers and the public of coccidioidomycosis, leading to more testing, as well as better availability of diagnostic tests offered by commercial laboratories.

A false-positive immunoglobulin M test result might lead to incorrect diagnosis of coccidioidomycosis if diagnosis is confirmed solely by this serological test (8). The positive predictive value of the enzyme immunoassay for coccidioidomycosis has been shown to vary depending on the circumstances under which the assay was used (9). In this study, those with history of travel to areas where coccidioidomycosis is endemic were significantly more likely to receive a diagnosis on the basis of positive culture, PCR testing, or both, compared with those without such travel, who were more likely to receive a diagnosis with serological tests. Because culture and PCR are more accurate tests for diagnosing recent coccidioidomycosis compared with the serological tests, whether all patients with no travel history were experiencing current infection is unknown. The coccidioidomycosis surveillance case definition makes no distinction between those with travel or residence in an area where coccidioidomycosis is known to be endemic and those without such a history, even though the history affects the positive predictive value of current diagnostic tests. Because persons living in areas without endemic disease have a much lower risk for having coccidioidomycosis, more stringent requirements for laboratory diagnosis of these cases might be prudent.

Four patients in the group that did not report travel received a diagnosis on the basis of positive coccidioidomycosis culture, raising the possibility that the disease was locally acquired. Soil analysis for *Coccidioides* spores in the area where those patients resided could have been helpful for clarification of whether the cases were truly locally acquired. Recently, *Coccidioides* was found in soil in south-central Washington, a state where coccidioidomycosis was not believed to be endemic; three acute coccidioidomycosis cases have been reported in Washington (10). No reports documenting the presence of *Coccidioides* spores in Missouri soil have been published in the indexed literature. Cluster analyses of a larger sample of coccidioidomycosis cases using software that analyzes spatial, temporal, and space-time data using spatial, temporal, or space-time scan statistics might be helpful for more accurate estimation of the possibility of endemic cases in Missouri.

The findings in this report are subject to one main limitation. The retrospective analysis was conducted on routine public health surveillance data, and no medical chart review or direct patient interviews were conducted. The surveillance data were not sufficiently complete in some cases with respect to demographics, travel history, medical history, clinical symptoms, diagnosis, treatment, and follow-up. In some cases, the exact diagnostic tests used for serology (e.g., immunoglobulin G or immunoglobulin M) or the exact titer for those tested by complement fixation were not known. Follow-up of patients with coccidioidomycosis to ensure that no alternative diagnoses emerged often was not available.

Epidemiology of coccidioidomycosis has been well described in states where it is known to be endemic, such as Arizona and California, but little information exists about it in other states. This research is a first attempt to study the epidemiology of coccidioidomycosis in a state without known endemic disease. Sustained surveillance for coccidioidomycosis in non-endemic states is important to ascertain whether locally acquired cases are occurring.

**Summary**

What is already known on this topic?

The incidence of reported coccidioidomycosis is increasing nationally, both in states where the disease is known to be endemic and those where it is not.

What is added by this report?

This is the first study of the epidemiology of coccidioidomycosis in a state without endemic disease. In Missouri, during 2004–2013, reported coccidioidomycosis incidence per 100,000 population significantly increased from 0.05 to 0.28. Nearly half of the patients with known travel history had visited areas where coccidioidomycosis is endemic, and were more likely to receive a diagnosis of the disease by fungal culture and polymerase chain reaction, rather than serological assays.

What are the implications for public health practice?

Surveillance for coccidioidomycosis is needed in non-endemic states to discover if locally acquired cases are occurring. For persons living in areas where coccidioidomycosis is not believed to be endemic, more stringent requirements for laboratory diagnosis of coccidioidomycosis might be appropriate.

## Figures and Tables

**FIGURE 1:**
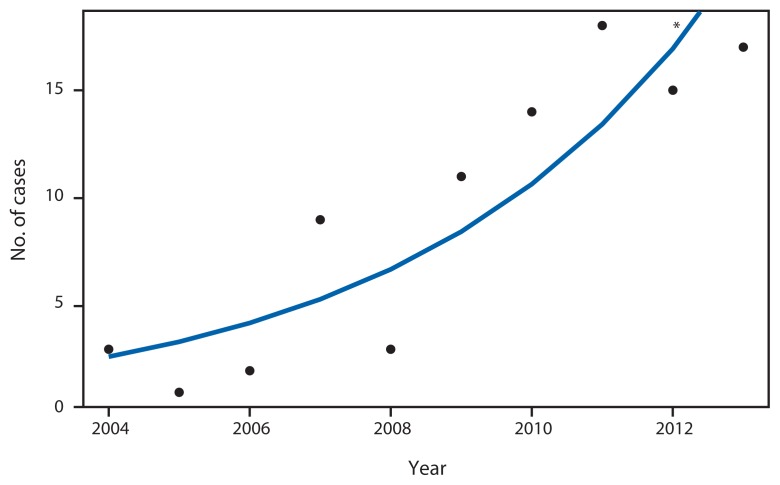
Incidence of coccidioidomycosis, by year — Missouri, 2004–2013 * Line represents estimated Poisson Regression model *y* =*e*^−463.29+0.23×^*^year^*; p<0.001.

**FIGURE 2:**
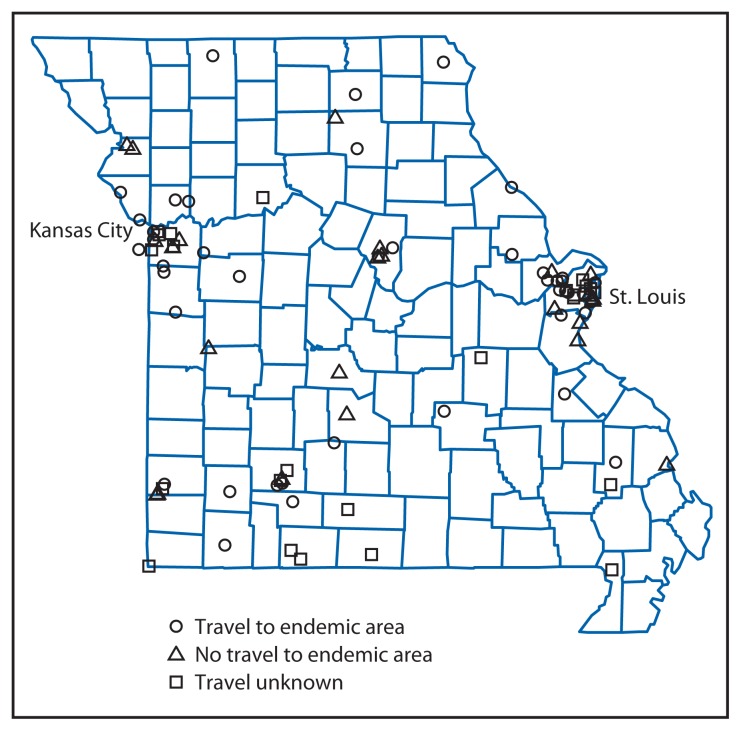
Coccidioidomycosis cases, by location and travel status — Missouri, 2004–2013

**TABLE:** Demographic and clinical characteristics of coccidioidomycosis cases — Missouri, 2004–2013

Characteristic	No.	(%)
**Total**	**93**	**(100)**
**Sex**		
Male	67	(72)
Female	26	(28)
**Age (yrs)**		
≥70	22	(24)
60–69	21	(23)
50–59	16	(17)
40–49	15	(16)
30–39	12	(13)
20–29	6	(6)
10–19	1	(1)
**Race**		
White	50	(54)
Black	7	(8)
Pacific Islander	1	(1)
Asian	1	(1)
Unknown	34	(37)
**Manifestations**		
Symptomatic lung lesions/Pneumonia	37	(40)
Flu-like illness	31	(33)
Hemoptysis	5	(5)
Headache/Confusion	3	(3)
Skin lesions	3	(3)
Sepsis/Disseminated	2	(2)
Asymptomatic lung lesions	2	(2)
Arthritis/Arthralgia	2	(2)
Meningitis	1	(1)
Unknown	7	(8)
**Laboratory tests**		
Culture	29	(31)
CF	28	(30)
Immunodiffusion	23	(25)
EIA/ELISA	13	(14)
PCR	4	(4)
Histopathology	2	(2)
Unknown serology	9	(10)

**Abbreviations:** CF = complement fixation; EIS/ELISA = enzyme immune assay/enzyme linked immunosorbent assay; PCR = polymerase chain reaction.
